# Methicillin-resistant staphylococcus aureus (MRSA) colonization among Intensive Care Unit (ICU) patients and health care workers at Muhimbili national hospital, Dar Es Salaam, Tanzania, 2012

**DOI:** 10.11604/pamj.2015.21.211.4207

**Published:** 2015-07-23

**Authors:** Alfred Geofrey, Ahmed Abade, Said Aboud

**Affiliations:** 1 Tanzania Field Epidemiology and Laboratory Training Program, Dar Es Salaam, Tanzania; 2 Muhimbili University of Health and Allied Sciences (MUHAS), Dar Es Salaam, Tanzania

**Keywords:** MRSA, colonization, prevalence, ICU

## Abstract

**Introduction:**

Methicillin-resistant Staphylococcus aureus (MRSA) has been recognized as important nosocomial pathogens worldwide. S aureus may induce clinically manifested diseases, or the host may remain completely asymptomatic.

**Methods:**

A cross-sectional hospital-based study was conducted from October 2012 to March 2013 in two ICUs at MNH. Admitted patients and health care workers were enrolled in the study. Interviewer administered questionnaires; patient history forms, observation charts and case report forms were used to collect data. Swabs (nostrils, axillary or wounds) were collected. MRSA were screened and confirmed using cefoxitin, oxacillin discs and oxacillin screen agar. Antibiotic susceptibility was performed using Kirby-Bauer disk diffusion method. The risk factors for MRSA were determined using the logistic regression analysis and a p - value of <0.05 was considered as statistically significant.

**Results:**

Of the 169 patients and 47 health workers who were recruited, the mean age was 43.4 years ± SD 15.3 and 37.7 years ± (SD) 11.44 respectively. Among the patients male contributed 108 (63.9%) while in health worker majority 39(83%) were females. The prevalence of MRSA colonization among patients and health care workers was 11.83% and 2.1% respectively. All (21) MRSA isolates were highly resistant to penicillin and erythromycin, and 17 (85.7%) were highly sensitive to vancomycin. Being male (AOR 6.74, 95% CI 1.31-34.76), history of sickness in past year (AOR 4.89, 95% CI 1.82- 13.12), being sick for more 3 times (AOR 8.91, 95% CI 2.32-34.20), being diabetic (AOR 4.87, 95% CI 1.55-15.36) and illicit drug use (AOR 10.18, 95%CI 1.36-76.52) were found to be independently associated with MRSA colonization.

**Conclusion:**

A study identified a high prevalence of MRSA colonization among patients admitted in the ICU. MRSA isolates were highly resistant to penicillin and erythromycin. History of illegal drug use was highly associated with MRSA colonization.

## Introduction

Globally, MRSA has been a common cause of infection in the hospital setting and it now accounts for more than 50% of staphylococcal infections in the community making its existence more important than ever [1]. When present in a host, S. aureus may induce clinically manifested diseases, or the host may remain completely asymptomatic; this condition is known as colonization [2]. These MRSA infections are generally seen in individuals who have ongoing interactions with the healthcare system for example dialysis patients and these infections may develop in these individuals as outpatients [3]. A study conducted at Muhimbili National Hospital (MNH) to assess the incidence of bloodstream infection and risk factors for fatal outcome of children aged zero to seven years with signs of systemic infection showed that 12% of hospital isolated S.aureus were MRSA. However, there was limited data on magnitude of MRSA and risk factors in Tanzania among patients admitted at the ICU and health care workers working in the facility. A study was conducted to determine the magnitude of and risk factors for MRSA colonization among ICU patients and health care workers at MNH, in Dar es Salaam.

## Methods

### Study design and setting

A cross-sectional hospital-based study was conducted from October 2012 to March 2013 in two ICUs at MNH in Dar es Salaam, Tanzania. MNH is a tertiary facility which receives referred patients from other health facilities all over the country. The ICUs admit serious sick patients of various underlying diseases who need close observation from the health care workers

### Study population

Patients admitted and health care workers attending the patients in two ICUs of MNH were included in the study. The health care personnel included physicians and nurses who have direct contact to admitted patients. Patients who had stayed for at least 24 hours at ICU were eligible for enrolment in the study. Only ICU health care workers or patients who gave the informed consent were enrolled in the study. All patients with prior staphylococcal infection were excluded from the study. The exclusion was achieved by referring to patient's history forms. A total of 216 participants (169 ICU patients and 47 health care workers) were enrolled in this study.

### Data collection and analysis

Interviewer administered questionnaires were used to collect socio-demographic and risk factors information for both health care workers and patients. The risk factors for MRSA colonization for patients included duration of stay in the hospital, prior antibiotic use, being diabetic, illicit drug use, being sick in the past one year and smoking habit for patients. The risk factors for health workers included profession, gloves exchange rate, hand washing and wearing surgical masks. Patient's history forms and observation charts were used to obtain more information of the patients. Laboratory result (case report) forms were used to collect laboratory results during diagnosis. The outcome variable was presence or absence of MRSA while the independent variables included age, sex, education level, occupation and risk factors. Epi Info version 3.5.1 (2008) was used for data entry and analysis. Descriptive statistics summarized the characteristics of patients and staff and their prevalence of MRSA. The risk factors for MRSA were determined using the logistic regression and a p - value of <0.05 was considered as statistically significant. To controlling the factors, the entire factor that was significantly associated and had a p ≤0.2 were subjected to model. Stepwise backward elimination unconditional logistic regression final model was use to come up with the final best model.

### Specimen collection

Swabs were collected from either of the following sites; anterior nostrils, axillary regions or wounds of the ICU patients and health care workers. Anterior nostrils were the principal sites for swab collection except for the patients inserted with nasal gastric (NG) tubes who were collected from either of the remaining mentioned sites above. The swabs were collected using sterile cotton swabs in the stuart transport medium and immediately transported to the laboratory for culturing.

### S. aureus isolation

All swabs were inoculated on blood agar medium containing 5% blood and 7.5% mannitol salt agar (Oxoid^®^, England) and incubated at 35°C for 24 hours [4]. S aureus was firstly identified by using colony morphology on 5% blood agar. Creamish to golden yellow colonies with or without hemolysis were further identified using Gram staining, a coagulase test, and a DNAse test (Oxoid^®^, England). In case of discrepancy between the coagulase and the DNAse tests, a latex agglutination test (Slidex Staph Test, England) was carried out [4].

### MRSA detection

MRSA detection was done using cefoxitin, oxacillin discs (Oxoid^®^) and oxacillin screen agar (5% NaCl, 6mg/ml oxacillin) [4]. Plates were incubated at 37 °C for cefoxitin disc and at 33 °C in the case of oxacillin disc and oxacillin agar. All isolates resistant to cefoxitin and oxacillin were considered as MRSA.

### Antimicrobial susceptibility testing (AST)

Kirby Bauer National Committee for Clinical Laboratory Standards (NCCLS) modified disk diffusion technique was used to determine antimicrobial susceptibility for MRSA. The antibiotics used were sulfamethoxazole, tetracycline, erythromycin, clindamycin, vancomycin and penicillin. Mueller Hinton sensitivity testing agar was inoculated with MRSA, antibiotics above were placed in a single plate and incubated at around 35 °C for 16 to 18 hours. Inhibition zone sizes were measured and interpreted using NCCLS standard S aureus ATCC 25923.

### Ethical considerations

The study was conducted according to the existing ethical guidelines. Ethical approval was obtained from the MUHAS Senate Research and Publications Committee. Written informed consents were obtained from the relatives of the admitted patients and health care workers at ICU. Participants who were found to carry MRSA were managed according antimicrobial susceptibility tests results returned to them and other clinical information since there is no treatment guideline MRSA cases at MNH.

## Results

**Figure 1:**
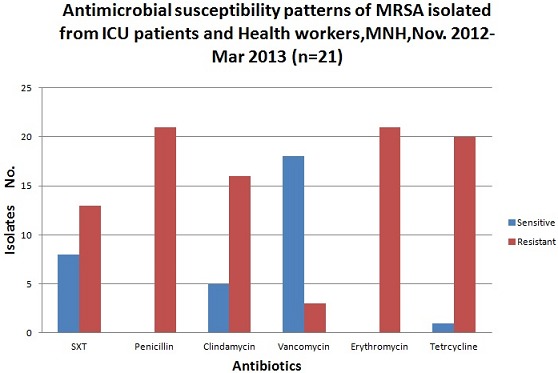
Antimicrobial susceptibility patterns of mrsa isolates

Of the 169 patients and 47 health workers who were recruited, the mean age was 43.4 years ± SD 15.3 and 37.7 years ± (SD) 11.4 respectively. Among the patients male contributed 108 (63.9%) while in health worker majority 39(83%) were females. Table 1 summarizes prevalence of MRSA colonization among patients by socio-demographic characteristics. The overall prevalence of MRSA colonization among patients was 11.83%. Of 20 MRSA isolates, 18 (90%) were from male. Patients with 60 years and above had the highest prevalence (23.5%) among the age groups Table 2 summarizes prevalence of MRSA colonization among patients by clinical characteristics. MRSA colonization was high among patients with muscular pains (33.3%) followed by fever (22.2%). Of 47 health care workers, only one was colonized with MRSA. The respondent was a male, 26 years old residing in Dar es Salaam with tertiary educational level and assistant nurse by profession All 21(100%) MRSA isolates were resistant to penicillin and erythromycin, and 16(76.2%) to clindamycin while majority 18(85.7%) were sensitive to vancomycin. The antimicrobial susceptibility patterns of the MRSA isolated from patients and health care workers from the ICU are shown in Figure 1. Being male and history of illicit drug uses were found to be independently associated with MRSA colonization.Table 3 summarizes the multivariate analysis of risk factors (socio-demographics) of MRSA colonization among patients admitted. Table 4 summarizes multivariate analysis of risk factors (clinical characteristics) of MRSA colonization among patients. History of sick in the past year, being sick more than three times, history of illicit drug use, skin infection and being diabetic were independently associated with MRSA colonization among patients.

**Table 1:** Prevalence of MRSA among patients (N=169) admitted at MNH ICU by socio-demographic characteristics

Characteristics	Category	n	MRSA		P-value
			+ ven(%)	-ve n (%)	
**Age (years)**	0 - 15	10	2 (20.0)	8 (80.0)	0.34
	16 - 30	22	2 (9.1)	20 (90.9)	0.5
	31 -45	57	5 (8.8)	52 (91.2)	0.38
	46 - 60	63	7 (11.1)	56 (88.9)	0.82
	60 and above	17	4 (23.5)	13 (76.5)	0.12
**Sex**	Male	108	18 (16.7)	90 (83.3)	0.01
	Female	61	2 (3.3)	59 (96.7)	0.01
**Education level**	Primary	108	12 (11.1)	96 (88.9)	0.7
	Secondary	45	5 (11.1)	40 (88.9)	0.86
	Tertiary	7	2 (28.6)	5 (71.4)	0.19
	No formal education	9	1 (11.1)	8 (88.9)	0.69
**Occupation**	Business	56	5 (8.9)	51(91.1)	0.41
	Farmer	30	3 (10.0)	27 (90.0)	0.51
	Health worker	7	2 (28.6)	5 (71.4)	0.19
	Retired	29	5 (17.2)	24 (82.8)	0.17
	Other	34	3 (8.8)	31 (91.2)	0.4
**Residence(Regions)**	Inside DSM	110	12(10.9)	98(89.1)	0.61
	Outside DSM	59	8(13.6)	51(83.3)	0.61

N = total respondents, n = frequency *(other regions of Tanzania)

**Table 2:** Prevalence of MRSA among patients (N=169) admitted at MNH ICU by clinical characteristics

Characteristic	n	MRSA		P-value
		+ ve n (%)	-ve n (%)	
Fever	9	2 (22.2)	6 (77.8)	0.24
Muscular pain	6	2 (33.3)	4 (66.7)	0.25
Confusion	18	1 (5.6)	17 (94.4)	0.34
Dizziness	4	1(25.0)	3 (75.0)	0.40
General body malaise	49	5 (10.2)	44 (89.8)	0.68
Respiratory symptoms	72	7 (9.7)	65 (90.3)	0.46
Chills	1	0 (0)	1(100)	0.88

**N = total respondents, n = frequency**

**Table 3:** Multivariate analysis+ of risk factors (socio -demographic) of MRSA colonization among patients (N=169) admitted at MNH ICU, Nov. 2012 to March 2013

Characteristic	COR	95% CI	AOR	95%CI
**Age**				
0 - 15	1.96	0.39 - 9.95		
16 - 30	0.72	0.15 - 3.33		
31 -45	0.62	0.21 - 1.81		
46 - 60	0.9	0.34 - 2.38		
60 and above	2.62	0.76 - 8.99		
**Sex**				
Male	5.9	1.32 - 26.38	6.74	1.31-34.76
female	0.17	0.03 - 0.8		
**Education**				
Primary	0.83	0.32 - 2.15		
Secondary	0.91	0.31 - 2.66		
Tertiary	3.2	0.58 - 17.72		
No formal education	0.93	0.11 - 7.83		
**Occupation**				
Business	0.64	0.22 - 1.86		
Farmer	0.8	0.22 - 2.91		
Health worker	3.2	0.58 - 17.72		
Other	0.67	0.19 - 2.44		
Retired	1.74	0.38 - 5.23		
**Smoking habit**				
Current smoker	5.5	0.62 - 41.22		
Ex-smoker	5.08	1.42 - 17.98	0.27	0.05 - 1.37
Non smoker				
**History of illegal drug use**				
Yes				
No	12.17	2.5 - 59.27	10.18	1.36-76.52

+ Only variables significantly associated with outcome were included in the final model COR = Crude odds ratio, AOR = adjusted odds ratio, CI confidence interval

**Table 4:** Multivariate analysis+ of risk factors (clinical characteristics) among patients (N=169) admitted at MNH ICU, Nov. 2012 to March 2013

Characteristics	Category	COR	95% CI	AOR	95%CI
Fever	Yes				
	++No	2.65	0.5 - 14.12		
Muscular pain	Yes				
	++No	4.03	0.69 - 23.57		
Confusion	Yes				
	++No	0.41	0.05 - 3.25		
Dizziness	Yes				
	++No	2.56	0.25 - 25.89		
Body weakness	Yes				
	++No	0.8	0.27 - 2.32		
chills	Yes				
	++No	0	undefined		
RS	Yes				
	++No	0.7	0.26 - 1.84		
Sick in past 1 year	Yes				
	++No	4.892	1.82 - 13.12	4.89	1.82 - 13.12
Number of times of sickness in past 1 year(either admitted or not)	++<3times				
	>3times	10.37	3.38 - 31.79	8.91	2.32 - 34.20
Patient with NG tubes	Yes				
	++No	0.9	0.35 - 2.20		
Source of admission	Hospital				
	++Home	0.66	0.18 - 2.58		
Used antibiotics (past 3 months)	Yes				
	++No	6.76	2.24 - 20.4	1.16	0.63 - 31.32
History of SSTIs	Yes				
	++No	7.9	2.16 - 29.17	7.9	2.16 - 29.17
Diabetes	Yes				
	++No	4.48	1.67 - 12.02	4.87	1.55 - 15.36
Cancer	Yes				
	++No	7.79	0.47 - 129.73		
Kidney	Yes				
	++No	0.86	0.18 - 4.05		
Heart diseases	Yes				
	++No	1.11	0.43 - 2.87		
Hypertension	Yes				
	++No	1.52	0.46 - 5.0		
Asthma	Yes				
	++No	1.25	0.14 - 10.99		
TB	Yes				
	++No	8.17	1.08 - 61.57	5.45	0.87 - 7.89
Bacterial infections(systemic)	Yes				
	++No	2.75	0.68 - 11.14		

+ Only variables significantly associated with outcome were included in the final model

++ =reference group, COR = Crude odds ratio, AOR = adjusted odds ratio, CI = confidence interval RS = respiratory symptoms

## Discussion

MRSA is the leading cause of nosocomial infections and are usually associated with poor outcomes in ICUs [5]. This study found the prevalence of MRSA colonization of 11.8% among patients admitted and 2.1% among health care workers in the same ICUs. MRSA isolates showed the highest resistance to erythromycin and penicillin. In multivariate analysis, being male, history of sickness in the previous year, illicit drug use, skin superficial infection, being sick more than three times in a past year and being diabetic were found to be independently associated with MRSA colonization among patients. In the current study we found a high prevalence (11.83%) of MRSA colonization among patients admitted in the ICU. This can be due to already colonized patients on admission in which transient hand carriage of the organisms on the hands of health care workers could account for the major mechanism for the patients-to-patients transmission [6]. This prevalence is comparable to the one found under 5-years children (10.5%) in Dar es Salaam, Tanzania (unpublished). A possible explanation for the slight higher prevalence includes differences in socio-demographic characteristics (age, occupation) and health conditions as former study was done to healthier individuals. It is also similar to that reported in previous studies in USA in 2004, 2007 and 2007 which ranged from 11.4% to 15.7% [7–9]. The finding differs with the prevalence of MRSA in eight countries that was reported to be relatively high (21-30%) in Nigeria, Kenya and Cameroon and low (10%) in Tunisia and Algeria [10]. The prevalence is different to 14.9% reported by Wang and his colleague in Taiwan and the difference could be due to differences in study design as they conducted a case control study [1].

MRSA isolates showed the highest resistance to penicillin and erythromycin. In the current study, disk diffusion technique was used to detect MRSA among S. aureus isolates. This resistance could be determined by the mecA gene, which encodes the low-affinity penicillin-binding protein PBP 2A [11]. The current study found that being male, history of sickness in the past year, illicit drug use, skin superficial infection and being diabetic contributed to the MRSA colonization in patients while admitted in the ICU. Being male and diabetes as independent risk factors of MRSA colonization found in this study have been reported in the previous studies [12, 13]. Diabetic patients are prone to MRSA colonization as they have reduced immunity which fails to combat the pathogens. The reason why male were more likely to be colonized with MRSA than female could be due to higher proportion (63.9%) of male than female recruited in the study. History of being sick in the past year and its association to MRSA colonization could be due to increased MRSA exposure in hospital settings when they were seeking treatment. The reason for association between skin superficial infections MRSA colonization could be due to weakened integrity of skin which reduce skin immunity to fight against MRSA. Patients with history of illicit drug use were more likely to be colonized with MRSA than non user. The reason for this could be due to the contamination of intravenous drug devices (e.g syringe) they share to inject drugs. The study has some limitations. It was not possible to analyze the risk factors of MRSA colonization among health care workers as only one staff was colonized. A cross sectional study was conducted in which patients were not screened prior to admission and not followed up to determine whether they got colonized before or during their stay in the ICU. It was not possible to conduct molecular typing to characterize MRSA due to limited budget.

## Conclusion

There is existence of MRSA colonization among ICU patients and health care workers. Of the risk factors, illegal drug use was highly associated with MRSA colonization among ICU patients. Patients should be screened prior to ICU admission to identify the one carrying MRSA. Antibiotic sensitivity tests are recommended for effective treatment of MRSA. Patients and health care workers should be screened prior to ICU admission and routinely, respectively, to identify individuals carrying MRSA. MRSA screening should prioritize patients at risk to MRSA colonization. Since MRSA has shown to be resistant the most commonly used antibiotics, the antibiotic susceptibility testing is recommended for effective treatment of MRSA.
